# Targeting pediatric sarcoma with a bispecific ligand immunotoxin targeting urokinase and epidermal growth factor receptors

**DOI:** 10.18632/oncotarget.21187

**Published:** 2017-09-23

**Authors:** Kristy Pilbeam, Hongbo Wang, Elizabeth Taras, Rachel J. Bergerson, Brianna Ettestad, Todd DeFor, Antonella Borgatti, Daniel A. Vallera, Michael R. Verneris

**Affiliations:** ^1^ Pediatric Blood and Marrow Transplantation, University of Minnesota, Minneapolis, MN 55345, USA; ^2^ Radiation Oncology, University of Minnesota, Minneapolis, MN 55345, USA; ^3^ Biomedical Statistics, University of Minnesota, Minneapolis, MN 55345, USA; ^4^ Veterinary Medicine, University of Minnesota, Minneapolis, MN 55345, USA

**Keywords:** Ewing’s sarcoma, rhabdomyosarcoma, urokinase-type plasminogen activator receptor (uPAR), epidermal growth factor receptor (EGFR), immunotherapy

## Abstract

Children with high risk sarcoma have a poor prognosis despite surgical resection, irradiation and chemotherapy. Alternative therapies are urgently needed. Urokinase-type plasminogen activator receptor (uPAR) and epidermal growth factor receptor (EGFR) are surface proteins expressed by some pediatric sarcomas. We show for the first time that a de-immunized bispecific ligand toxin, EGFATFKDEL, directed against EGFR and uPAR, successfully targets pediatric sarcoma. Using flow cytometry, we identified a rhabdomyosarcoma (RMS) cell line, RH30, that expresses both uPAR and EGFR, and a Ewing sarcoma (EWS) cell line, TC-71, that expresses only uPAR. We tested the differential sensitivity of these two sarcoma cell lines to toxin-induced killing, using both *in vitro* assays and an *in vivo* murine model. We show that pediatric sarcomas are highly sensitive to EGFATFKDEL (at subnanomolar concentrations) *in vitro*. *In vivo*, tumor growth was significantly attenuated after treatment with EGFTFKDEL, compared to untreated controls, in both RH30 and TC-71 tumor bearing mice. In addition, we found that simultaneously targeting both receptors in a dual positive cell line was more effective than targeting a single receptor or antigen, resulting in a greater tumor response, including complete tumor regression in an animal model of bulky disease. Our findings provide support for further exploration of bispecific targeting of pediatric sarcomas with bispecific ligand toxins, such as EGFATFKDEL.

## INTRODUCTION

Pediatric patients with localized, standard risk sarcoma have an ∼70% chance of long-term survival with modern treatment modalities [1]. In contrast, outcomes for high-risk patients with metastatic, relapsed or refractory disease are poor with a <30% chance of 5-year survival [1, 2]. Current multimodality sarcoma therapy includes chemotherapy along with surgery and/or radiation for local control. However, these therapeutic efforts may already be maximized, in terms of both benefit and toxicity. Clearly new and effective targeted agents, with an acceptable safety profile, are needed to improve the survival of patients with high-risk disease.

Urokinase-type plasminogen activator receptor (uPAR) is a glycosylphosphatidylinositol (GPI)-anchored cell surface protein that is associated with tumor cell invasion, migration and metastasis [3]. When uPAR binds to its ligand, urokinase-type plasminogen activator (uPA), plasminogen coverts to plasmin, facilitating extracellular matrix degradation and cancer cell invasion [3]. uPAR and uPA are overexpressed in many cancers, however, expression is best characterized in breast and pancreatic cancers, where it is associated with tumor progression [3, 4]. uPAR is found on both the tumor cell surface, as well as the tumor neovasculature, making it an attractive therapeutic target. mRNA expression of uPA system components has been detected in adult soft tissue sarcoma samples (n=78) [5], but little is known about uPA and uPAR in pediatric sarcomas. uPA mRNA has been identified in some rhabdomyosarcoma (RMS) cell lines and primary tumor samples, likewise uPA antigen expression has been detected in RMS cell lines [6, 7]. Expression of uPA mRNA has also been shown in Ewing sarcoma (EWS) cell lines and in some primary patient samples [7].

Aberrant epidermal growth factor receptor (EGFR) expression and signaling promotes the proliferation, migration and invasion of malignant cells, and is frequently associated with metastasis and an overall poor prognosis [8]. EGFR is overexpressed, mutated, or dysregulated in numerous cancers and hence, is an attractive target for many malignancies. While targeting EGFR has been widely explored in adult solid tumors, the effectiveness of EGFR targeting in pediatric sarcomas is less clear [9–11]. EGFR has been detected at varying levels in both cell lines and tumor samples of the three most common pediatric sarcomas, RMS, EWS and osteosarcoma (OS) [9–14]. Efforts to target EGFR with monoclonal antibodies (cetuximab) and small molecule inhibitors (gefitinib and erlotinib) have been extensively investigated in adult cancers leading to FDA approval for some EGFR overexpressing cancers [15–18]. These same therapies have shown some pre-clinical benefit in pediatric sarcomas [14, 19] and while phase I/II clinical trials demonstrated safety, they provided little clinical benefit [20–22]. Cetuximab, an EGFR antibody, has been used preclinically on both RMS and OS cell lines [10, 12, 13], but this drug showed little benefit in clinical trials [23]. Given that the mechanism of these agents is to interrupt EGFR signaling that leads to cell growth and resistance to apoptosis, one interpretation of the above data is that while some pediatric sarcomas express EGFR (and EGFR family member receptors), they do not exclusively rely upon them for growth and survival. However, these findings do not preclude the use of this receptor for therapeutic drug delivery.

Collectively, the above data suggests that uPAR and EGFR are attractive receptors to target on pediatric sarcomas, but the existing agents are not clinically effective. Theoretically, targeting cancer with a bispecific agent should be more effective than a monospecific agent, but this is largely unexplored. We previously created a bispecific, de-immunized immunotoxin (EGFATFKDEL) that targets EGFR on tumor cells and uPAR, both on tumor cells and their neovasculature [24–26]. This bispecific ligand toxin was de-immunized by mutating seven immunodominant B-cell epitopes on the pseudomonas exotoxin, PE38, to reduce immunogenicity without loss of efficacy [25]. EGFATFKDEL has been effective against both head and neck carcinomas and glioblastoma in preclinical murine models [25–27]. More recently this bispecific immunotoxin has been tested in a canine trial of hemangiosarcoma [28]. Here we show for the first time that a targeted bispecific ligand immunotoxin, EGFATFKDEL, is effective at killing pediatric sarcomas both *in vitro* and *in vivo* using immune-deficient murine models of established disease, giving promise to this agent as a potential future therapy. Moreover, we demonstrate the relative benefit of dual antigen targeting with a bispecific immune toxin.

## RESULTS

### Expression of EGFR and uPAR

**Figure 1:**
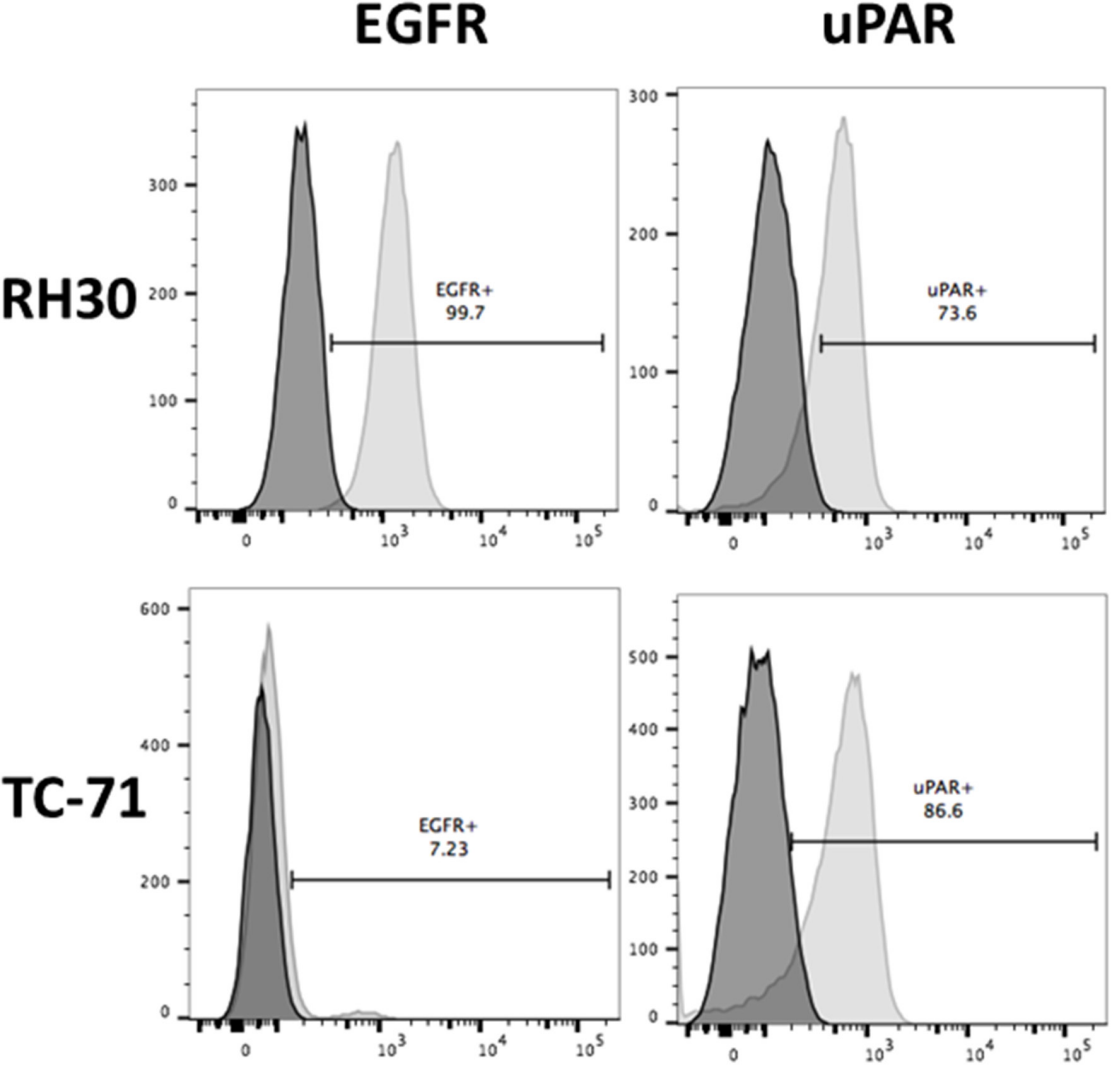
Surface expression of EGFR and uPAR on RH30 (RMS) and TC-71 (EWS) cell lines by flow cytometry Cells lines were stained with mAbs against EGFR Brilliant Violet 421 and uPAR APC. Representative experiment is shown with 73.6% and 86.6% of RH30 and TC-71 showing uPAR expression and 99.7% of RH30 showing EGFR expression with 7.23% on TC-71 cells. The x-axis on the right represents uPAR surface expression and on the left represents EGFR surface expression. The dark shaded plot represents the unstained control sample while the lighter shaded plot represents the stained samples for each cell line.

Flow cytometry was used to determine the expression of both EGFR and uPAR on pediatric sarcoma cells derived from RMS (RH30) and EWS (TC-71). Surface staining for each cell line is shown in Figure 1. uPAR is highly expressed on both cell lines, while strong EGFR expression is seen only in RH30 cells. Thus, we identified two pediatric sarcoma cell lines that express uPAR, but differed in EGFR expression, allowing us to test the relative benefit of dual antigen targeting vs. monospecific targeting with the bispecific immunotoxin EGFATFKDEL.

### *In vitro* activity of EGFAFTKDEL on pediatric sarcoma cells

**Figure 2:**
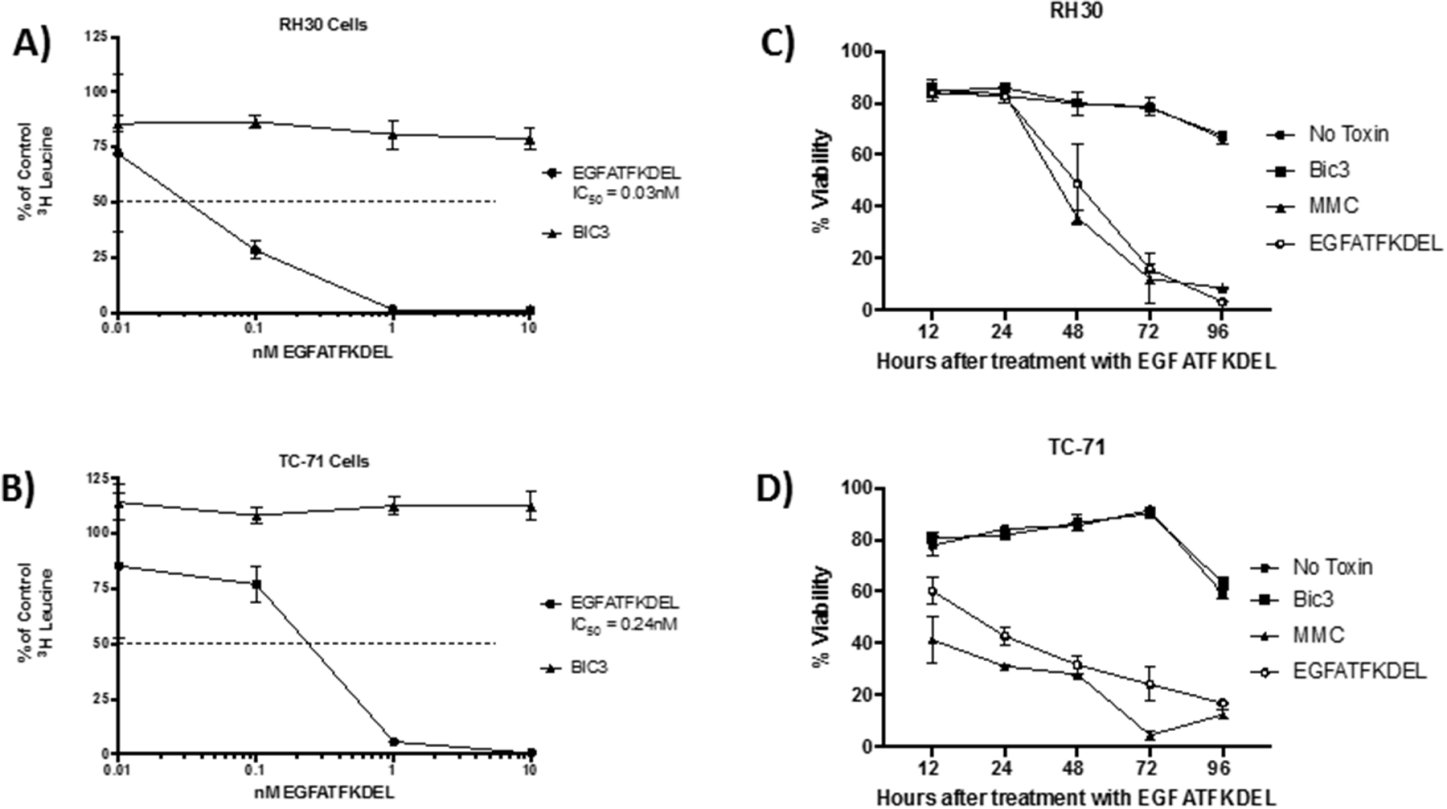
The effect of EGFATFKDEL on RH30 and TC-71 cells *in vitro* **(A** and **B)** ^3^H Leucine protein synthesis assay where 1x10^4^ cells/ well were plated in triplicate and allowed to adhere overnight. RH30 (A) or TC-71 (B) cells were then pulsed for 72 hours with increasing doses (0.01-100 nM) of either EGFATFKDEL (circle) vs. BIC3 (triangle, negative control). ^3^H Leucine uptake was measured and data are reported as percent control response (y-axis). **(C** and **D)** shows the percent viability using Annexin V and 7AAD staining of RH30 (C) and TC-71 cells (D) treated with 2.5 μg EGFATFKDEL (open circle), compared to mitomycin positive control (triangle). Negative controls included BIC3 treated (square) and untreated cells (closed circle). Cells were harvested and analyzed at time points ranging from 12-96 hours. Above experiments are representative of 2 individual experiments.

To determine the activity of EGFATFKDEL on both pediatric sarcoma cell lines, we performed functional studies using a ^3^H-leucine incorporation assay (to measure protein synthesis) and annexin V and 7-AAD staining (to determine apoptosis and cell death). Figure 2 shows the effect of EGFATFKDEL on both sarcoma cell lines using these assays. EGFATFKDEL effectively halts protein synthesis at subnanamolar concentrations. However, the IC_50_ values were 6 times lower for RH30 cells (that express both uPAR and EGFR) when compared to TC-71 cells (that express only uPAR; 0.04 nM vs. 0.24 nM, Figure 2A and 2B). Next, we examined the ability of EGFATFKDEL to induce cell death in the two cell lines. Figure 2C and 2D shows the percent viability for both RH30 and TC-71 cells at progressive time points after treatment with EGFATFDEL Controls included no treatment, CD3 targeted immunotoxin (BIC3, a negative control) and mitomycin C (MMC, positive control). While the initial response to MMC differed over the first 24 hours, with TC-71 being more sensitive, both cell lines were largely apoptotic by 96 hours. Treatment with EGFATFKDEL yielded nearly identical results and kinetics to MMC (Figure 2C and 2D). Thus, both lines underwent apoptosis over the course of 96 hours with the bispecific ligand immunotoxin which was 3-7 times greater than untreated cells and irrelevant controls.

### Effect of EGFATFKDEL on tumor spheres

**Figure 3:**
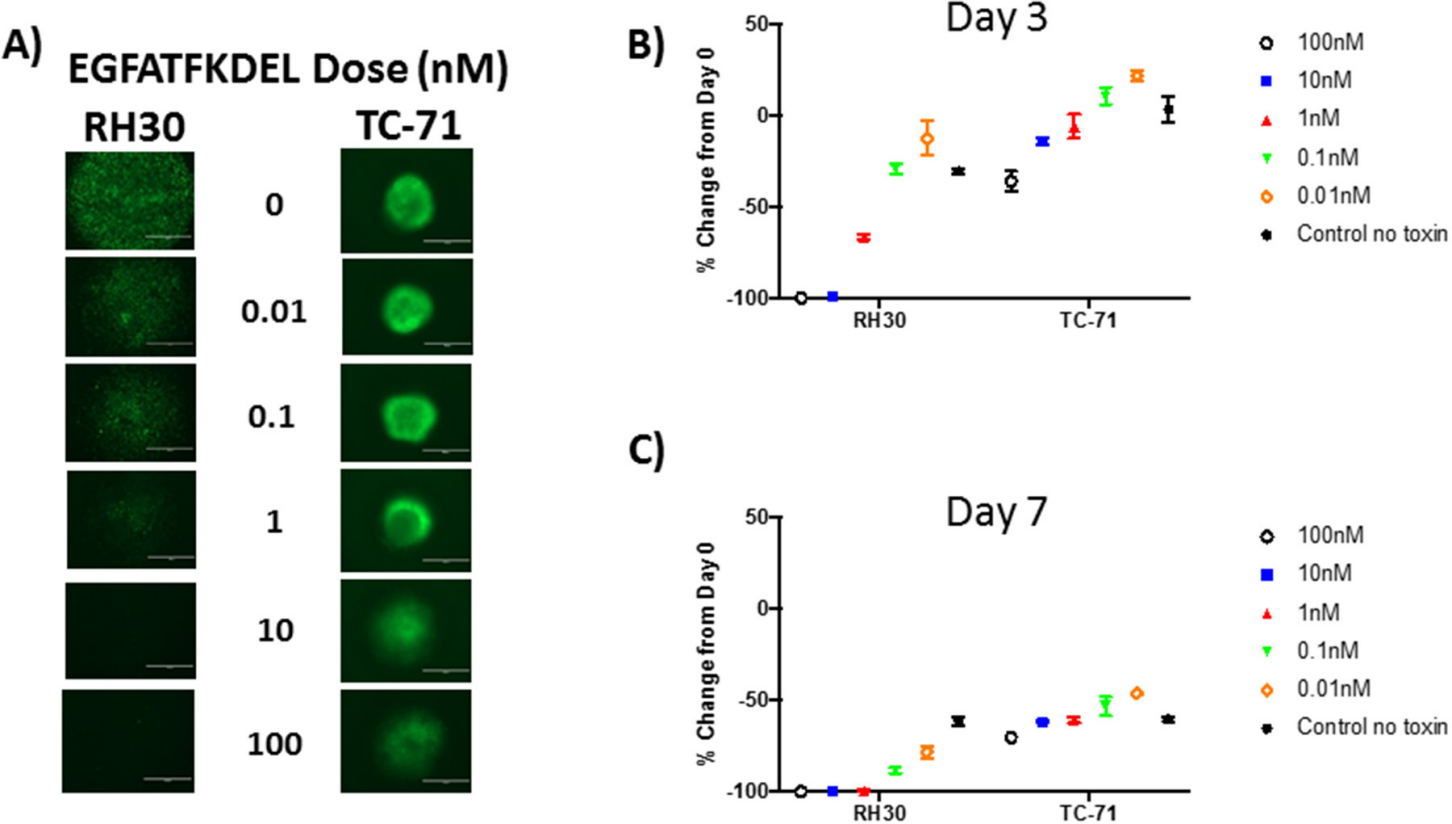
EGFATFKDEL treated RH30 and TC-71 tumor spheres **(A)** shows representative GFP images for RH30 (left) or TC-71 (right) tumor spheres, 3 days after treatment with EGFATFKDEL at doses ranging from 0-100 nM of. Data in **(B-C)** is representative of 2 experiments done in triplicate. (B) shows % change in GFP expression at day 3 for RH30 and TC-71 spheres and (C) shows the % change in GFP expression for day 7. Comparison in percent change of GFP expression between these two cell lines at these time points were significantly different (p <0.0001) with RH30 cells showing a greater response.

To simulate a three-dimensional tumor microenvironment, GFP-expressing RH30 and TC-71 cells were grown in a ULA round bottom plate over 10 days, allowing tumor sphere formation. Tumor spheres were then treated with 10-fold dilutions of EGFATFKDEL (0.01-100 nM) and to track viability, cells were monitored every 24 hours for loss of GFP expression by fluorescent microscopy, indicating cell death. This method provided a more physiologic model than treating a monolayer or cells and was used to evaluate the ability of EGFATFKDEL to penetrate a dense tumor mass with a single treatment. A representative series of images, three days post treatment, are shown in Figure 3A. At three and seven days, both RH30 and TC-71 cells showed a dose-dependent and progressive loss of GFP signal compared to untreated controls (Figure 3B and 3C). However, comparing the GFP signal at three days after treatment for RH30 (which expressed both EGFR and uPAR) to TC-71 (which only expressed uPAR) showed differences in GFP loss at 100 nM (100% loss vs 36%), 10 nM (65% loss vs 14%) and at 1 nM (36% loss vs 6%) (Figure 3B and 3C, p<0.001 for all). Similar differences between RH30 and TC-71 persisted at day 7 when cells were treated at 100 nM (100% loss vs. 70%), 10 nM (88% loss vs. 62%), 1 nM (78% loss vs. 61%), 0.1 nM (88% loss vs. 53%) and 0.01 nM (78% loss vs 46%) (p<0.001 for all).

### Treatment of RH30 and TC-71 tumor bearing mice with EGFATFKDEL

**Figure 4:**
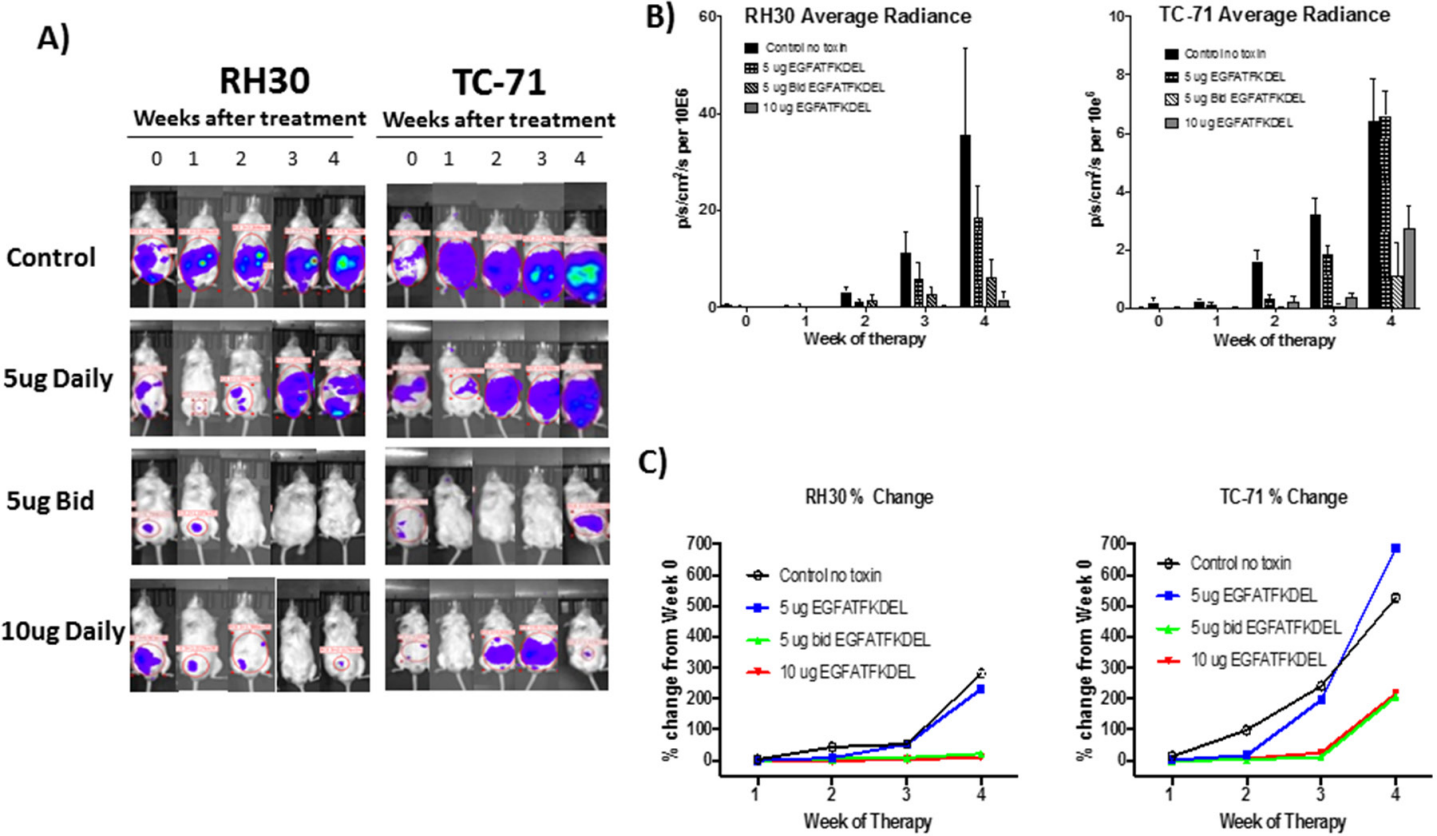
*In vivo* tumor models **(A)** Representative RH30 and TC-71 tumor bearing mice treated with EGFATFKDEL compared to untreated control mice. **(B)** Average radiance of all RH30 (left, n=24) and TC-71 (right, n=34)-bearing mice, as measured by BLI. Mice were treated with stated dose (5μg daily/5μg BID/10μg daily on M, T, Th, F for 4 weeks). **(C)** Average % change in the average radiance of RH30 (left, n=24) and TC-71 (right, n=34)-bearing mice over 4 weeks of treatment compared to controls.

To further test the efficacy of mono-specific vs. bispecific targeting of EGFR and uPAR, we established murine models of RMS and EWS using luciferase-transduced RH30 and TC-71 cells lines, respectively. Mice were treated with EGFATFKDEL or PBS (control) and monitored weekly using BLI to measure therapeutic response. Figure 4A shows representative mice for each group over time. All control mice (no treatment) showed tumor progression. In contrast, the majority of RH30 and TC-71 bearing mice showed EGFATFKDEL-induced tumor regression (67% and 58% respectively). Overall, few mice in this established tumor model showed sustained complete responses, however significantly more of the bispecific targeted RH30 tumor bearing mice vs. monospecific targeted TC-71 mice did achieve this (17% vs 0%, p=0.04). The average radiance of luciferase over time, which is indicative of tumor burden, is shown in Figure 4B for all mice in the various treatment groups, demonstrating a dose dependent response to EGFATFKDEL for both RH30 and TC-71. We also examined the percent change of BLI average radiance from baseline in each group over time (Figure 4C). When compared to controls, both RH30 and TC-71 tumor bearing mice treated with EGFATFKDEL at 5 μg BID and 10 μg daily showed significantly less tumor growth (5μg BID p=0.02 and <0.01; 10μg daily p <0.01, =0.01). Interestingly, when we compared the percent change in tumor growth between the RH30 and TC-71 cells, there was a significantly better response in the dual targeted RH30 tumors after 4 weeks (p=0.02), suggesting that targeting both receptors is more efficacious *in vivo*.

## DISCUSSION

The purpose of this study was to determine whether EGFATFKDEL could be used in pediatric sarcoma. These studies show for the first time that a bispecific ligand toxin targeted to EGFR and uPAR can successfully treat pediatric sarcoma in a pre-clinical model. Efforts to target EGFR in pediatric sarcoma have been attempted previously, with little success when translated to patients [10–12, 14, 19–23] while uPAR has not previously been targeted in pediatric sarcoma. Our bispecific ligand toxin represents a novel approach to targeting pediatric sarcoma by utilizing an overexpressed receptor to deposit a de-immunized pseudomonas exotoxin within the cancer cell, rather than blocking a receptor or signaling pathway.

A secondary aim of this work was to determine whether dual targeting was more effective than targeting a single receptor. EGFKDEL and ATFKDEL monospecific ligand toxins previously have been shown to be inferior to the bispecific targeting agent, EGFATFKDEL, in inducing cell death in neoplastic cells [25]. The specificity of these toxins was confirmed using a blocking assay as well as treating EGFR- uPAR- neoplastic cells [25]. Here, we identified a RMS line, RH30, that expressed both EGFR and uPAR and a EWS line, TC-71, that lacked EGFR expression, but expressed uPAR. We demonstrate both *in vitro* and *in vivo* that EGFATFKDEL is effective at killing pediatric sarcoma cells. Additionally, our results show that dual targeting of these receptors appears to be more efficacious and maybe superior to monospecific targeted therapies. Therefore, this bispecific immunotoxin potentially represents a novel treatment approach to pediatric sarcoma.

Prior studies show that the pseudomonas toxin possesses strong anticancer activity and interrupts protein synthesis [25]. We show that in pediatric sarcoma cells treated with pseudomonas toxin, there is an arrest in protein synthesis and an induction of apoptosis over the course of 96 hours. Both cell lines were sensitive to EGFATFKDEL at subnanomolar levels, however based on the IC50, ∼6-fold higher doses were required to halt protein synthesis in EWS cells which only express uPAR. In line with this data, we show that the immunotoxin is more effective in killing RH30 tumor spheres, with significant cell death apparent at 72 hours, while in contrast, TC-71 tumor spheres showed diminished, but persistent GFP signal at both 72 hours and 7 days. A similar study of canine hemangiosarcoma, which express both EGFR and uPAR, showed that EGFATFKDEL effectively killed both hemangiosarcoma cells in monolayer as well as hemangiospheres enriched for cancer stem cells at subnanamolar doses over 72 hours [29].

Similar to the *in vitro* studies, we used a rigorous murine model of established disease to test whether the dual immunotoxin was more effective in treating cells expressing both receptors (RH30), compared to a line expressing only uPAR (TC-71). In both tumors, the mice showed significant dose-dependent tumor regressions in response to EGFATFKDEL treatment. Collectively, our data show that mice bearing tumors expressing both EGFR and uPAR (i.e., RH30) had a greater effect on tumor growth over time, along with a higher number of complete responses to the toxin, compared to the TC-71 bearing mice that only expressed uPAR. As above, we interpret these data to suggest that bispecific targeting is more effective than monospecific targeting, but fully acknowledge that differential sensitivity of each cell line to the toxin is also possible. Future studies will use shRNA to knock-down each receptor in RH30 cells to more precisely address this topic in a single cell line. EGFATFKDEL, has been effective when delivered intra- tumorally in a murine model of glioblastoma flank tumors [25], however our model here shows efficacy by IP injection in a more disseminated cancer model. Similar IP injections of a bispecific ligand toxin (BLT) toxin targeting EGFR and IL-4R successfully treated metastatic breast cancer in mice [30].

We did not observe any of the commonly reported side effects seen with EGFR inhibitors, such as cutaneous eruptions and GI toxicity [31–33], likely because EGFATFKDEL does not interfere with EGFR signaling per se and instead, uses EGFR to deliver a de-immunized pseudomonas toxin to cells. Overall, the mice tolerated treatment with EGFATFKDEL well. Mild weight loss was observed in both tumor groups by the 3^rd^ week of therapy for mice treated with 5 μg BID and 10 μg daily. Treatment doses were held for weight loss >10%, resulting in resolution of weight loss in those mice. At the end of the study several mice showed ascites and bowel distension. These findings are consistent with similar murine models of BLTs that have reported weight loss and mild hepatotoxicity at higher doses when given intraperitoneally [30]. Not surprisingly, higher doses of BLTs have been well tolerated when administered intra-tumorally, while subcutaneous delivery at even lower doses leads to significant dermatologic reactions [26, 30]. In the canine trials that used intravenous EGFATFKDEL for companion dogs with hemangionsarcoma (expressing both EGFR and uPAR), cutaneous or GI toxicities were not observed and EGFATFKDEL was overall well tolerated. The canine trial did report reversible hypotension during the immunotoxin infusion that was subsequently improved with pre-hydration and additional fluid boluses. Thus, local irritation from repeated IP injection likely explains the findings in our murine studies. While mouse gender should not affect sarcoma growth or response to the toxin, our study only included male mice so that we could match age and treatment group size at the time of the study. As well, the mice used for these studies were immune deficient and while the pseudomonas immunotoxin was deimmunized, the effect of the immune system on tumor responses seems unlikely given the canine data.

The results of the phase I canine trials are promising, showing significantly prolonged survival in dogs receiving standard of care surgery and chemotherapy + EGFATFKDEL versus standard of care alone [28]. Importantly, these companion dogs with naturally-occurring hemangiosarcoma had low stage disease and were treated in the microscopic disease setting following splenectomy. Thus, when added to traditional chemotherapy, EGFATFKDEL is clearly active in the setting of low disease burden. Our *in vivo* model takes an important next step, showing that EGFATFKDEL is also active in aggressive, bulky tumors even when administered as a single agent. We speculate that like the canine studies, this drug would be most active in patients with micro-metastatic disease and in combination with chemotherapy. While prior efforts to block EGFR on pediatric sarcoma have had limited success in inducing tumor regression and preventing progression, we show that receptors that are selectively overexpressed on tumor cells can be used to deposit a pseudomonas exotoxin, leading to tumor cell death. Moreover, our studies suggest that bispecific targeting is more effective than monospecific targeting, perhaps providing more specificity. This study provides support for further investigation of this bispecific ligand toxin in the setting of metastatic, relapsed and refractory pediatric sarcomas, as well as other cancers that express these receptors.

## MATERIALS AND METHODS

### Cell lines

Human pediatric cell lines included those from RMS (RH30) and EWS (TC-71), both were gifted from the Children’s Oncology Group (COG) prior to this work and a certificate of authentication was provided. All cell lines were certified mycoplasma negative, reconfirmed by our laboratory using PCR and authenticated. All cell lines were maintained using the following media: RPMI-1640 (RH30) and Iscove’s modified Dulbecco’s medium (TC-71) supplemented with 10% fetal bovine serum, 2 mmol/l L-glutamine, 100 units/ml penicillin, and 100 μg/ml streptomycin. All cells were grown in culture flasks in a humidified 37°C atmosphere containing 5% CO_2_. Cells were passaged using trypsin- EDTA when they reached ∼ 80–90% confluence. Only cells with viability >95%, as determined by trypan blue exclusion, were used for experiments. Transfected cells (see below) were maintained in culture and periodically (every 4 weeks minimum) cultured with zeocin (100 μg/ml) for TC-71 and RH30 cells.

### Mammalian cell transfection

Each of the above cell lines were transfected with a green fluorescent protein (GFP) and luciferase-expressing plasmid containing a zeocin resistance gene using a transposon-transposase system. The transposon and transposase plasmids were gifted from Dan Kauffman (University of Minnesota Masonic Cancer Center). Transfection was performed by electroporation using the Amaxa V kit from Lonza (Basel, Switzerland) (RH30) or lipofectamine 3000 from Thermo Fisher Scientific (Waltham, MA), (TC-71). Once transfected, cells were treated with a lethal dose of zeocin to select for a population of transfected cells. The appropriate zeocin concentrations were determined individually for each cell line. Transfection was confirmed using GFP expression by both flow cytometry and fluorescent microscopy. Luciferase expression was also confirmed with bioluminescence imaging.

### Surface expression

EGFR and uPAR surface expression was assessed using flow cytometry. The following antibodies were purchased from BioLegend (San Diego, CA) and eBioscience (San Diego, CA) respectfully: EGFR Brilliant Violet 421 and uPAR APC. Cells were stained on ice for 30 min and were then analyzed on an LSR flow cytometer at the University of Minnesota Masonic Cancer Research Center core facility and analyzed with flowjo software.

### EGFATFKDEL bispecific immunotoxin

Using DNA shuffling and cloning techniques we produced a single chimeric gene that coded for a single-chain bispecific ligand immunotoxin, known as EGFATFKDEL [25]. This gene consists of segments from the following human genes: epidermal growth factor (EGF) and amino terminal fragment (ATF) of uPA linked to the first 362 amino acids of the pseudomonas exotoxin A (PE38). PE38 was modified by adding Lys-Asp-Glu-Leu (KDEL) C-terminus signal that prevents secretion of the protein from the ER and enhances anticancer activity. This molecule was de-immunized after prior testing established improved targeting and reduced immunogenicity with mutation of 7 amino acids of interest. A construct of this gene is shown in Supplementary Figure 1 and cloning and production is previously described in detail in [25, 26].

### Leucine protein synthesis assay

^3^H-Leucine protein synthesis assays were used to measure the effect of EGFATFKDEL on sarcoma cell protein synthesis, which is an indirect measure of proliferation and viability *in vitro*. BIC3, a PE38KDEL containing immunotoxin that targets CD3 expressed on T cells was used as a negative control [34]. 1x10^4^ cells/ well were plated in a 96 well flat bottom culture plate in leucine free media and allowed to adhere overnight at 37°C with 5% CO_2_. Cells were treated with 10-fold dilutions (0.01 nM -100 nM) of either EGFATFKDEL or BIC3 in triplicate. Cells were incubated for 72 hours at 37°C with 5% CO_2_ and ^3^H-Leucine was added 24 hours prior to harvest. The plates were frozen to detach cells and then allowed to thaw for 1 hour prior to harvesting onto a glass fiber filter. Filters were then washed, dried, and counted using standard scintillation methods. Background counts in untreated wells ranged from <10-500 CPM. Data are reported as percentage of background control counts.

### Apoptosis assays

Annexin V and 7AAD staining was performed to confirm that EGFATFKDEL induced apoptosis and cell death. Briefly, 2.5 x 10^5^ cells/ well were plated in 6 well flat bottom culture plates and incubated overnight at 37°C with 5% CO_2._ When cells were at ∼75% confluence, they were treated with 2.5 μg of EGFATFKDEL and harvested at 12, 24, 48, 72 and 96 hours after treatment. BIC3 (2.5 μg) was used as a negative control and 0.01 mg/ml of Mitomycin C was used as a positive control. Adherent cells were harvested with StemPro Accutase from Thermo Fisher Scientific (Waltham, MA) at the above times and were stained using Annexin V PE and 7-AAD following manufactures protocol from BD. Cells were analyzed by flow cytometry on a Canto flow cytometer and analyzed with flowjo software.

### Tumor sphere cytotoxicity assay

RH30 and TC-71 cells were plated at 1000 cells/ well in 96 well round bottom ultra-low attachment (ULA) plates. Spheres established over 10 days. EGFATFKDEL was added in 10-fold dilutions (0.01 nM-100 nM) along with untreated controls. Spheres were evaluated for loss of GFP expression every 24 hours for 7 days using identical parameters and settings with a EVOS FL Life Technologies GFP microscope. A consistent ROI was used and GFP signal was quantified using ImageJ software.

### Immunodeficient murine sarcoma models

Male NSG mice (NOD.Cg-Prkdc^scid^Il2rg^tm1Wjl^/SzJ) between 10-12 weeks old were used for each experiment. Mice were housed in an Association for Assessment and Accreditation of Laboratory Animal Care-accredited specific pathogen-free facility under the care of the Department of Research Animal Resources, University of Minnesota. Animal research protocols were approved by the University of Minnesota Institutional Animal Care and Use Committee. All animals were housed in microisolator cages to minimize the potential risk of infection. Mice were age matched within each study and ear notched for identification. Briefly, mice were inoculated with 5 x 10^5^ (TC-71) or 3.75 x 10^5^ (RH30) tumor cells by Intraperitoneal (IP) injection. Tumors were allowed to establish over 5 days and were confirmed by bioluminescence imaging (BLI) prior to treatment. Mice were randomly assigned to treatment or control groups. EGFATFKDEL was administered by IP injection in 100μl of sterile saline 4 days a week (Mon/Tue/Thur/Fri) for a total of 4 consecutive weeks. Treatment groups included the following doses of toxin: 5μg daily, 5μg twice daily (BID) or 10μg daily. Sterile PBS was injected as a control. Mice were imaged weekly in an IVIS Spectrum *In vivo* imaging system to measure tumor growth and/or response to treatment. A change in BLI was based on the average radiance in the ROI over time when compared to day 5 (BLI at study entry). Tumor regression was defined as a decrease in BLI from baseline measured at day 5 prior to the start of treatment. Complete response was defined as undetectable signal by BLI. To image tumors, mice were given 100 μl of a 30 mg/ml D-luciferin aqueous solution (Gold Biotechnology, St Louis MO) as a substrate for luciferase by IP injection ∼10 minutes prior to imaging. Isoflourine gas was used for mouse sedation during imaging and data was analyzed using living imaging software. Identical ROIs were established for each mouse and consistently used at each imaging time point for the duration of the experiment. Therapy related toxicity was monitored with bi-weekly weight measurements.

### Statistics

For *in vitro* analysis, a two way ANOVA was used to calculate p-values. In the murine studies, repeated measures using Mixed Models Analysis longitudinally were used to compare the percent change in radiance from baseline to 4 weeks testing the hypothesis that mean values of the logarithm of the percent change in flux were different from each other. Tukey’s adjusted p-values were used in comparing across treatment groups, if the overall p-value was significant, to adjust for multiple comparisons. To evaluate the binary endpoints of response (change less than baseline) and complete response (value of 0) over time, longitudinal non-linear mixed models were used to assess a logistic regression model controlling for correlation of within mouse measures assuming a normally distributed random variance component (McMahon et al. 2006). All-reported p-values are 2-sided. Proc Mixed from SAS version 9.3 was used for all analysis.

## SUPPLEMENTARY MATERIALS FIGURE



## Abbreviations

(uPAR): Urokinase-type plasminogen activator receptor
(EGFR): epidermal growth factor receptor
(RMS): rhabdomyosarcoma
(EWS): Ewing sarcoma
(OS): osteosarcoma
(MMC): mitomycin C
(BLT): bispecific ligand toxin
(GFP): green fluorescent protein
(IP): intraperitoneal
(BLI): bioluminescence imaging
