# Supplementary material for: Targeting pediatric sarcoma with a bispecific ligand immunotoxin targeting urokinase and epidermal growth factor receptors
Source: Oncotarget. 2017 Sep 23;9(15):11938–47. doi: 10.18632/oncotarget.21187 (PMC5844719; doi:10.18632/oncotarget.21187)
Supplement: Supplementary file 1 [file oncotarget-09-11938-s001.pdf]

Targeting pediatric sarcoma with a bispecific ligand immunotoxin targeting urokinase and epidermal growth factor receptors

SUPPLEMENTARY MATERIALS

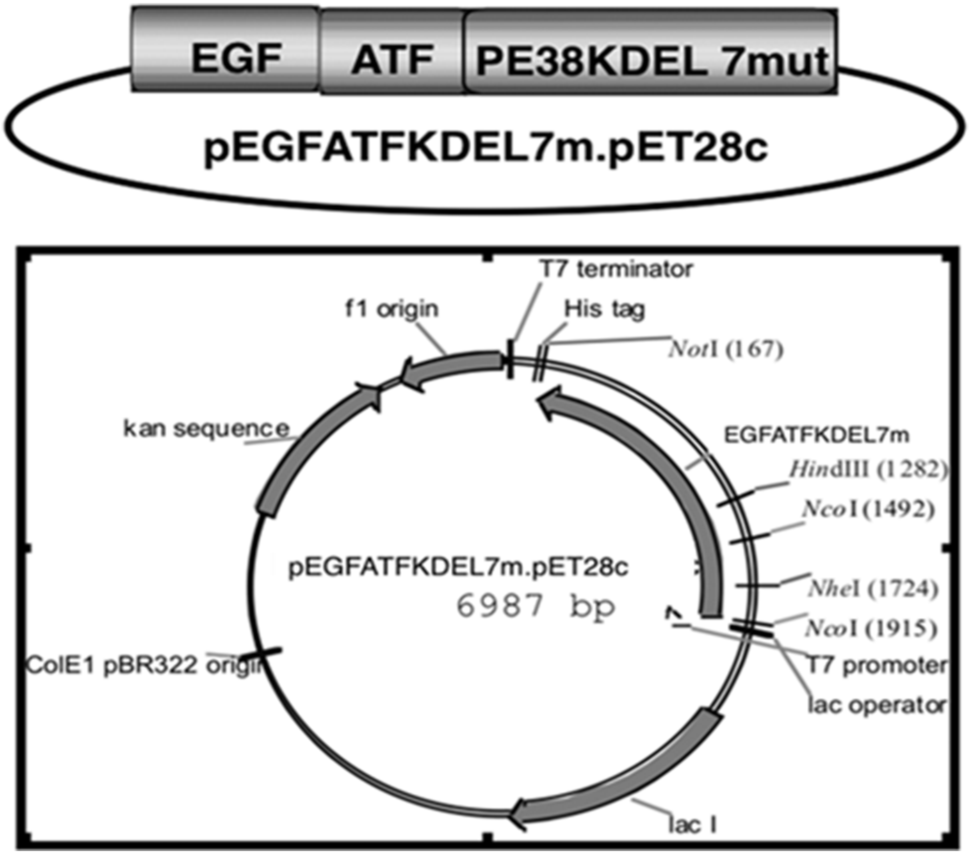

Supplementary Figure 1: Construct of de-immunized EGFATFKDEL. Schematic plasmid design and map where human EGF and the amino terminal fragment of urokinase were linked to a de-immunized PE38KDEL molecule (pseudomonas exotoxin).
